# Single-Cell Lipidomics by LC-MS Interlaboratory Study Reveals the Impact of X‑ray Irradiation on a Pancreatic Cancer Cell Line and Its Bystanders

**DOI:** 10.1021/acs.analchem.5c02010

**Published:** 2025-06-18

**Authors:** Kyle D. G. Saunders, Johanna von Gerichten, Rahul Deshpande, Matt Spick, Susan Bird, Giuseppe Schettino, Hannah Bolland, Anthony Whetton, Eirini Velliou, Melanie J. Bailey

**Affiliations:** † School of Chemistry and Chemical Engineering, Faculty of Engineering and Physical Sciences, 3660 University of Surrey, Guildford GU2 7XH, U.K.; ‡ 486281 Thermo Fisher Scientific, 355 River Oaks Parkway, San Jose, California 95134, United States; § School of Health Sciences, Faculty of Health and Medical Sciences, 3660 University of Surrey, Guildford GU2 7XH, U.K.; ∥ 9917 National Physical Laboratory, Hampton Road, Teddington TW11 0LW, U.K.; ⊥ School of Veterinary Medicine, Faculty of Health and Medical Sciences, 3660 University of Surrey, Guildford GU2 7XH, U.K.; # Division of Surgery and Interventional Science, 4919 University College London, London W1W 7TY, U.K.; 7 Department of Infectious Diseases, Guy’s Hospital, King’s College London, London SE1 9RT, U.K.

## Abstract

Live single-cell lipidomics by liquid chromatography mass spectrometry (LC-MS) is a nascent and rapidly growing field which can shed new light on infectious diseases, cancer, immunology, and drug delivery. There are now a growing number of laboratories that can isolate single cells and laboratories that can perform lipidomics analysis at correspondingly low sample volumes, but there is a lack of validation data. We have carried out the first interlaboratory LC-MS lipidomics experiment for single cells, aimed at filling this gap. We present a novel workflow to enable interlaboratory studies, comprising live-cell imaging and single-cell isolation, followed by freeze-drying, international shipping, reconstitution, and untargeted lipidomics analysis. We applied this methodology to reveal radiation-induced bystander effects in pancreatic cancer cells. X-ray irradiated cells and their bystanders sampled live 48 h postirradiation demonstrated reduced lipid abundance compared to controls, with distinct changes in molar ratios of several polyunsaturated lipids. This demonstrates for the first time that radiation can cause considerable cellular lipid remodelling, not only at the site of delivery. A striking similarity in lipid changes was observed between the two participating laboratories despite differences in sample preparation and analysis methods. Our results are further corroborated by live-cell imaging analysis of lipid droplets. This work serves as an important validation and demonstration of the nascent and rapidly growing field of live single-cell lipidomics.

## Graphical abstract

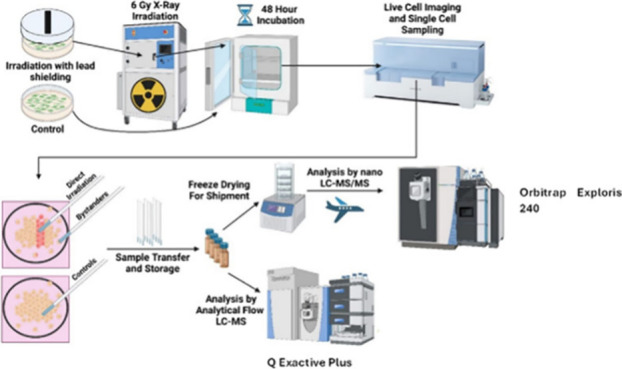

## Introduction

Lipids are essential for cellular function and affect cell shape, membrane formation, and function. As such, lipidomics is highly desirable to understand and mitigate conditions such as cancer, infectious diseases, diabetes, and cardiovascular disease. Since cell populations are highly heterogeneous, single-cell lipidomics is an attractive approach to understand basic biological mechanisms, information which may be lost when samples are studied in bulk. Mass spectrometry imaging approaches have emerged as an attractive solution for the rapid analysis of lipids at the single-cell level; however, they carry the disadvantage that cells are typically fixed prior to analysis and so are not sampled in their native state.^1^ In addition to this, there can be significant challenges in confident identification of lipids due to the overlap of isobaric and isomeric species.^2^

Single-cell lipidomics using liquid chromatography mass spectrometry (LC-MS) is a new field with only a handful of published papers.^3,4^ In contrast to mass spectrometry imaging approaches, living cells can be sampled in their native environment using either capillaries or microfluidics.^5–7^ Additionally, analytes are separated prior to ionization, which can reduce matrix effects, and peak annotation confidence can be improved through retention time matching.^8^ However, due to the very low volume of the cell, which is just a few pL, performing lipidomics by LC-MS on a single cell faces several challenges including sensitivity, the possibility to introduce contamination from solvents and other consumables, and a lack of validation and replication data.^9^ To address these limitations, the pursuit of new analytical methods is desirable, but equipment and expertise for cell culture, single-cell isolation, and high-end mass spectrometry are not necessarily colocated. As such, transporting extracted single-cell samples between laboratories for LC-MS lipidomics analysis is highly desirable but has not been reported, as the quantity of the sample is considerably less than that for bulk lipidomics such as plasma or whole-tissue samples. Furthermore, many lipid species are prone to oxidative stress and degradation, which must be considered in data interpretation.^10^ Here we propose a novel workflow to enable single-cell samples to be transported in LC-MS vials between laboratories to enable method development and validation.

Lipidomics also comes with significant challenges in confident structural identification due to the overlap of isobaric species and regioisomers, which may fragment similarly under standard collision induced dissociation (CID).^2^ To aid peak assignment, it is common for samples to be measured in both positive and negative modes to collect orthogonal fragmentation data. This is especially useful in combination with chromatography, allowing positive and negative mode fragments to be matched to the same lipid by retention time, increasing the confidence in the identification. Analysis in both ionization modes also can provide greater coverage of the lipidome, as some lipid classes do not ionize well in both modes.^11^ This is difficult to apply to single cells using LC-MS, as the sample volume is limited to avoid overdilution and typically only allows for a single injection, with some exceptions in single-cell proteomics and multiomics.^12,13^ Polarity switching allows positive and negative ions to be analyzed from the same injection and has been successfully demonstrated in untargeted serum lipidomics.^14^ As only one injection is required, polarity switching presents a potential opportunity to increase both the coverage and annotation confidence of lipids in a single cell. Here we show for the first time how polarity switching can aid lipid annotation and coverage in single cells.

The potential biological applications of single-cell lipidomics are significant and include gaining a detailed understanding of the development and treatment of infectious diseases, immune disorders, and cancer. In this work, we show how single-cell lipidomics can shed new light on radiation biology, specifically the radiation-induced bystander effect (RIBE). RIBE describes the influence of irradiation on unirradiated neighboring cells and healthy tissue, prompting DNA damage and cell death.^15^ This is of particular relevance to cancer radiotherapy, as wider systemic damage and even secondary cancers can arise from these downstream effects.^16^ RIBE has been associated with oxidative stress to off-target tissues, and given the association between oxidative stress and lipids, lipidomics is a suitable tool to study RIBE.^13^ Indeed, polyunsaturated fatty acid metabolism was previously implicated in the response to radiation injury and RIBE in a variety of cell lines, mice, and nonhuman primate models under experimental conditions.^17–19^ However, these studies have been conducted in serum or bulk cell extracts and, therefore, have not explored spatial or temporal relationships. Polyunsaturated lipids are of particular importance to the onset of ferroptosis, a recently discovered nonapoptotic mode of cell death characterized by uncontrolled iron-dependent lipid peroxidation.^20^ Here, we show how single-cell lipidomics can be used to explore the radiation induced bystander effect spatially and temporally in a cell culture model. The methodology involves selected area irradiation of a cancer cell monolayer, followed by live-cell imaging, capillary sampling of selected live cells (on/off target radiation), and LC-MS lipidomics analysis of lipids in single cells.

In this work, cells were divided into two groups after sampling; a discovery group that was shipped internationally for analysis, and a validation group that was analyzed in-house. A striking similarity in radiation-induced lipid markers was observed between the two participating laboratories. Parallel observations using confocal microscopy on the same cells analyzed by LC-MS corroborated these results. This work therefore serves as a validation of performing single-cell lipidomics in this way, as it shows for the first time that two laboratories performing LC-MS analysis of single-cell lipids can detect a similar biological effect despite differences in sample preparation and analysis methods.

## Methods and Materials

### Chemicals and Reagents

An isotopically labeled lipid standard mix EquiSPLASH (Avanti Polar Lipids, cat. no. 330731) was purchased from Sigma-Aldrich for use as a multiclass internal standard. Chromatography solvents (isopropyl alcohol (IPA), acetonitrile (ACN), and water) were Optima LC-MS grade and purchased from Fisher Scientific. Chloroform used for lipid extraction was HPLC grade (+99.5%) and also purchased from Fisher Scientific. Nile red, BODIPY 493/503, and Hoechst was purchased from Fisher Scientific for fluorescence imaging (cat. nos. N1142, D3922, and 34580, respectively). Dulbecco’s phosphate buffered saline (DPBS) was purchased from Sigma-Aldrich (cat. no. D8537). The cell culture media was prepared as described by Wishart et al.^44^ Dulbecco’s modified Eagle medium (DMEM) with high glucose (Sigma-Aldrich, Merck, UK, cat. no. 21969035) was supplemented with 10% (v/v) fetal bovine serum (Fisher Scientific, UK, cat. no 11550356), 1% (v/v) penicillin/streptomycin (Fisher Scientific, UK, cat. no. 15140122), and 2 mM l-glutamine (Sigma-Aldrich, Merck, UK, cat. no 25030024).

### Cell Culture

Human pancreatic adenocarcinoma cells (PANC-1, Merck, UK) were used for single-cell and cell extract measurements. Cells were cultured in Corning T25 culture flasks (Merck, UK) in DMEM prepared as described above. Cells were kept at 37 °C with 21% O_2_ and 5% CO_2_. Cell culture media were replaced on alternating days, and cells were passaged approximately twice a week, when confluency reached 80–90%. Prior to single-cell sampling, approximately 100,000 cells were seeded into BioLite cell culture treated dishes (Fisher Scientific, UK, cat no 11844335). The same volume of cell culture media (no cells) was simultaneously aliquoted into cell culture dishes to serve as a negative control.

### Cell Irradiation and Time Course Imaging

For time course imaging, cell culture dishes at ∼80% confluency were sealed with parafilm and placed in an Xstrahl CIX-2 Irradiator cabinet (Xstrahl, UK). Cells were irradiated at a distance of 30 cm from the source (with a 0.5 mm thick copper filter, first half value layer (HVL) = 1.56 mm) at 195 kV, 10 mA for 346 s, delivering a uniform dose of 6 Gy. Control cells were placed outside of the cabinet and received no dosage. Cells were then placed back in the incubator to re-equilibrate for 90 min before media were replaced with warmed 2 mmol/mL Nile red in FBS-free media to stain lipid droplets. Cells were then placed in the incubator of a Cellome SS2000 confocal microscope (Yokogawa, Japan), which was equilibrated to 37 °C and 5% CO_2_ with humidity control activated. Images were generated using a UPLXAPO 40x/0.95 dry (WD 0.18) objective and 8 μm z-stack projection for brightfield and fluorescence imaging (Ex 480 nm/Em 617 ± 73 nm) every 2 h for 48 h.

### Cell Irradiation and Sampling

Cell culture dishes at ∼80% confluency were sealed with parafilm, covered with a 21 mm thick lead shield with a distinct 4 × 21 mm rectangular slit through the center (pictured in Figure S1), and placed in an Xstrahl CIX-2 irradiator cabinet (Xstrahl, UK). Cells were irradiated using the same conditions described above. A dish of control cells was placed outside of the cabinet for the same amount of time and received no dose. Due to the lead cover, cells either side of the rectangular slit were shielded from radiation on either side of the irradiation zone. The efficacy of the lead shielding was assessed by placing EBT3 Gafchromic film (Gafchromic, USA) under the culture dish during the course of irradiation (Supplementary Figures S8 and S9). After irradiation, cells were placed back in the incubator for 48 h prior to sampling. Before single-cell sampling, cells were treated with 10 μM BODIPY 493/503 and 0.2 μM Hoechst 34580 for 30 min and washed again before the addition of FBS-free media.

For sampling, cells were placed into a Cellome SS2000 instrument (Yokogawa, Japan) with the incubator equilibrated, and cells were sampled according to the protocol described in previous work^24^ with radiation and bystander cells samples as shown in Supplementary Figure S10. Prior to single-cell sampling, cells were imaged with a 40× lens described above but with a 9 μm z-stack projection and fluorescence parameters to match Hoechst (Ex 405 nm/Em 447 ± 60 nm) and BODIPY (Ex 488 nm/Em 525 ± 50 nm). Therefore, fluorescent imaging data of lipid droplets were obtained for the cells actually sampled for downstream mass spectrometry. Cells were sampled from the “directly irradiated” and “bystander” zones as well as from a “control” dish, which received no dose. Negative controls were taken by aspirating media from a cell-free Petri dish into 10 μm capillaries.

### Transport Preparation

All cells and negative controls were placed on dry ice immediately after sampling and then transferred to a −80 °C freezer for storage. Of the cells collected, a “Discovery Group”, comprising 12 cells from each treatment condition (directly irradiated, bystander, control) were eluted from the sampling capillaries into QSert LC-MS vials with 5 μL of internal standard (EquiSPLASH, 16 ng/mL) according to the previously described protocol.^24^ This portion of the sampled cells was then freeze-dried in a FreeZone 2.5 L benchtop freeze-dryer (Labcono, USA) under low vacuum of 0.5 mbar and stored under nitrogen. Freeze-dried cells were then shipped from the United Kingdom to Thermo Fisher, California, at room temperature before receipt and storage at −80 °C. The remaining cells (12 from each treatment condition) comprised a “Validation Group” and were analyzed in-house by analytical flow LC-MS according to the protocol previously described.^25^ These cells were simply stored at −80 °C and eluted on the day of analysis. LC-MS conditions for this analysis can be found in Supplementary Table S1.

### Cell Dye Impact Assessment

To understand the potential impact of the live-cell imaging dyes on lipid metabolism and the mass spectrometry signal, single cells dyed with Hoechst and BODIPY using the same staining protocol as the irradiation experiment were sampled under the same conditions in the SS2000. In parallel, control cells that were under the same incubation conditions but without dye were also sampled. These cells were stored at −80 °C until the day of analysis before being eluted into LC-MS vials as described above and analyzed using the analytical flow LC-MS methodology.

### Nanoflow LC-MS/MS

The freeze-dried and shipped cells were analyzed using nanoflow LC-MS/MS. On the day of analysis, LC-MS vials were removed from −80 °C storage and reconstituted with 7 μL of IPA before being vortexed and sonicated. 7 μL of H_2_O was added to each vial, and 12 μL of each sample was injected onto the column. The analysis was conducted on a Vanquish Neo UHPLC (Thermo Fisher, USA) coupled to an Orbitrap Exploris 240 (Thermo Fisher, USA). Data were acquired with MS1 resolution of 60,000 and a *m*/*z* window of 250–1250. DD-MS2 was performed at 15,000 resolution, taking the top 4 scans, and HCD was set to 35. Polarity switching was used to obtain both positive and negative spectra, switching between polarities every 10 scans.

Chromatographic separation was achieved using an EASY-Spray PepMap column (75 μm × 15 cm) (Thermo Fisher, USA) running at 750 nL/min at 45 °C. Mobile phase A was 60:40 ACN/H_2_O, and mobile phase B was 88:10:2 IPA/ACN/H_2_O. Both mobile phases were supplemented with 10 mM ammonium formate and 0.1% formic acid. The gradient was 20 min in total and started at 70% A for 1 min, decreased to 45% A for 2 min, decreased further to 25% A for 2 min, decreased again to 5% A for 4 min, decreased to 0% A for 3 min, remained isocratic for 4 min, and then increased to 70% A for 0.2 min, remaining isocratic for 3.8 min. LC-MS/MS parameters are described in detail in Supplementary Tables S1–S3.

### Data Analysis and Statistics

MS-Dial (ver. 5.3.240719) was used to process the raw LC-MS/MS data by using a mass tolerance of 0.01 da for MS^1^ and 0.025 Da for MS^2^ to collect the data.^45^ A minimum amplitude of 10,000 was used to detect peaks with a mass slice width of 0.1 da. Smoothing was carried out with a linear weighted moving average using a scan smoothing level of 3 and a minimum peak width of 5 scans. Peak identification was carried out on the nano-LC-MS/MS data against the LipidBlast database with a mass tolerance of 0.01 Da for MS^1^ and 0.025 Da for MS^2^, with an 80% identification score cutoff. [M + H]^+^, [M + NH_4_]^+^, and [M + H – H_2_O]^+^ were allowed as adducts for identification. A tolerance of 0.1 min and 0.025 Da (MS^1^) was in place for peak alignment. Gap filling by compulsion was disabled for this analysis. Internal standards were used to calculate the mass of each lipid per single cell semiquantitatively, from which mol % values were calculated. Specifically, LPC, Cer, DG, PC, PE, PI, PG, PS, SM, TG, and CE were used. Identified lipids in the nano-LC-MS/MS data set were manually inspected to confirm the validity of identification using both positive and negative mode data obtained using polarity switching. This list of 211 high-confidence lipids was used to build an in-house library. Subsequent data generated with analytical flow LC-MS on the QExactive Plus was analyzed in MS-Dial using the same conditions as above, but identifications were made against the in-house library.

Multivariate analysis was conducted using the one factor statistical analysis function of Metaboanalyst.^46^ By default, missing values were replaced by 1/5 of the minimum positive values of their corresponding variables. No filtering was used within the analysis pipeline. Normalization was done in Excel using class-matched internal standards; therefore, no normalization was applied in MetaboAnalyst. Data were log transformed (base 10) and auto scaling was applied. To assess the validity of PLS-DA models generated in MetaboAnalyst, leave-one-out cross validation was applied to the first 5 components, and the *Q* ^2^/*R* ^2^ values were used to evaluate model performance. Freestyle (Thermo Scientific) was used for manual peak integration and inspection of MS spectra *ad hoc*. Analysis of the microscopy images were analyzed using ImageJ and CellPathfinder (Yokogawa). Two-tailed Mann–Whitney U tests were performed on the lipid droplet data using SPSS statistics (ver. 29.0.1.0) to determine statistical significance of lipid droplet size and frequency, with a *p*-value threshold of <0.05 set for significance. Significance of lipidomics data by Mann–Whitney U testing was calculated using GraphPad’s statistical analysis functions, with p-value thresholds of <0.05 and <0.01 set for significance and noted as * and ** in each figure, respectively.

A data-driven approach to test the similarity of bystander cells to either irradiated cells or control cells was used in this work by using a machine learning classification model. This model was implemented using a support vector machine with a linear kernel and a *C* (regularization factor) of 1. The Python code used to perform the analysis used the scikit-learn library without modification.^47^

## Results and Discussion

A schematic illustrating the irradiation, sampling, and analysis workflow is presented in Figure 1.

**Figure 1:**
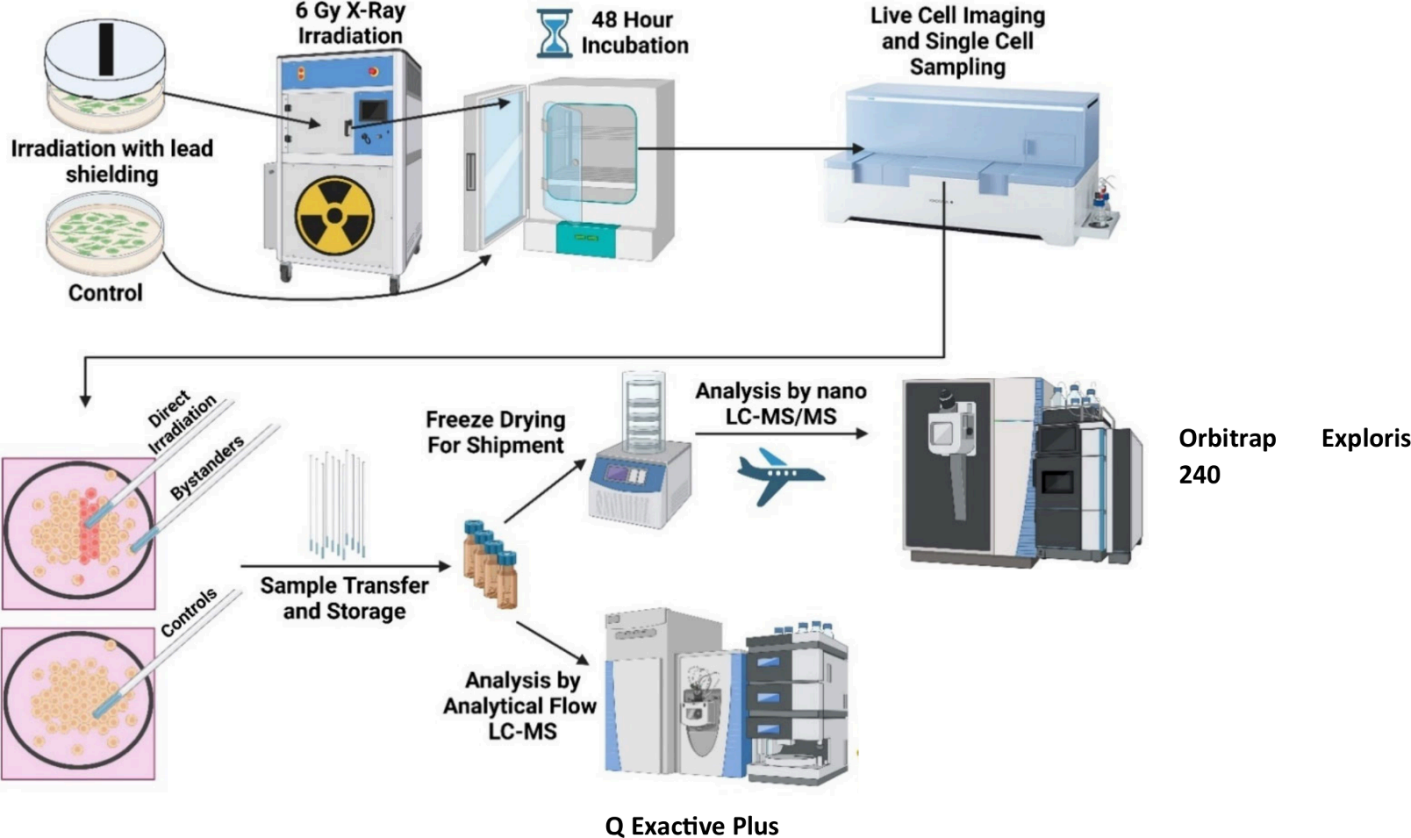
Workflow for cell irradiation, sampling, and analysis. PANC1 cells were irradiated through a slit in lead shielding and incubated for 48 h. The Yokogawa SS2000 was used to sample directly irradiated cells, bystanders, and cells from a control dish. The cells were divided into two batches: the first batch was stored at −80 °C and then analyzed by analytical flow LC-MS using an Orbitrap Q Exactive Plus instrument, and the second batch was freeze-dried, shipped at room temperature to San Jose, and analyzed using a nanoflow LC-MS method on an Orbitrap Exploris 240 instrument.

### Class Composition of Internationally Shipped Single Cell Lipids

Cells were dyed with Hoechst to identify the nucleus for single-cell location and BODIPY to visualize the lipid droplets formed under stress conditions. To understand the potential impact of these dyes used for imaging on both lipid metabolism and ion suppression, the lipid profiles from cells dyed with Hoechst/BODIPY were compared by principal components analysis, and Mann–Whitney U tests were conducted on all lipids between classes (Supplementary Figures S1 and S2 and Table S8). No significant difference was found for any lipid between the two groups, indicating a minimal impact on the observed lipid profiles by use of these live-cell imaging dyes.

The coverage, composition, variability, and consistency of detection of lipids in single PANC-1 cells after shipping are summarized in Figure 2 A–D, respectively.

**Figure 2:**
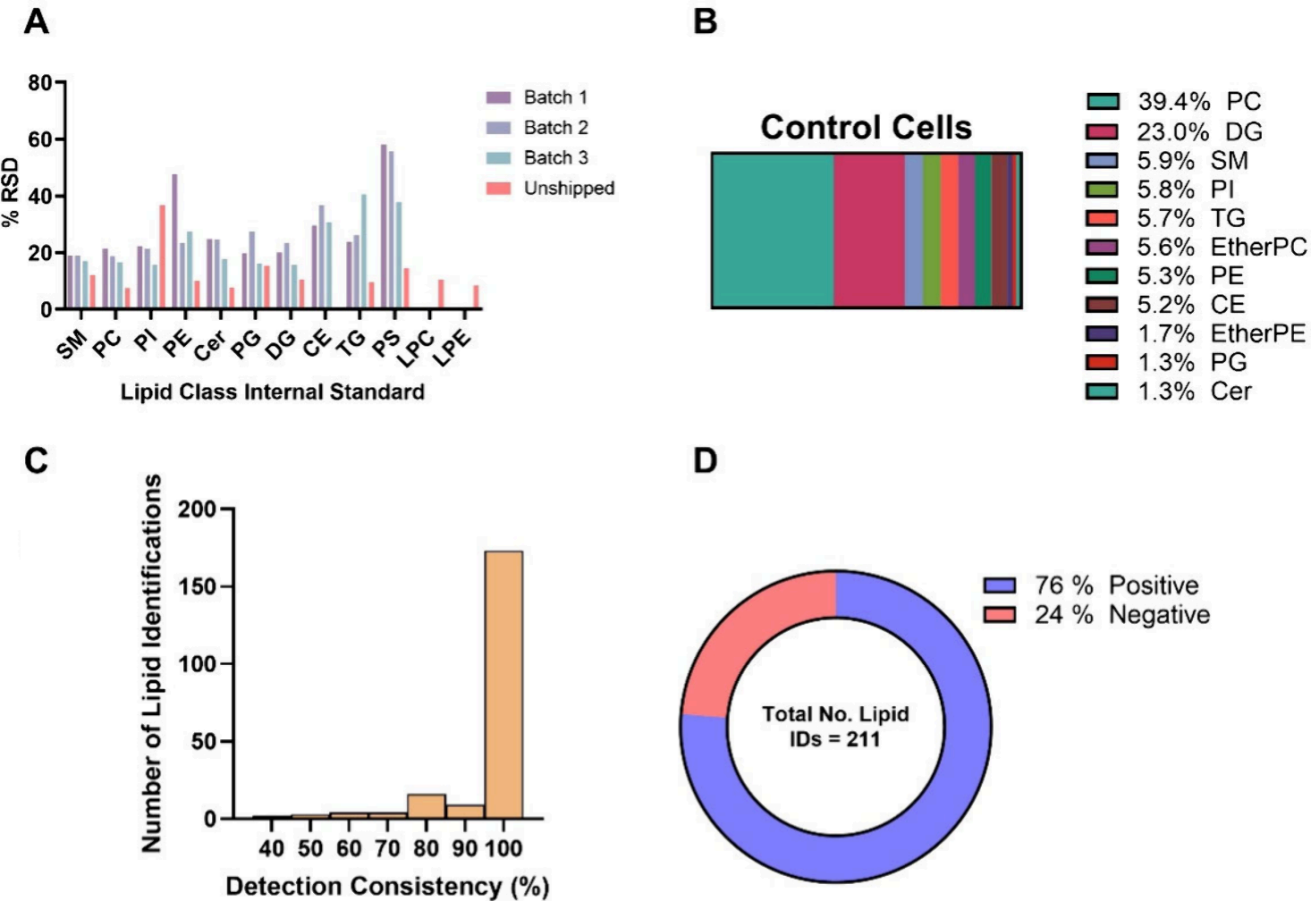
Single-cell lipidomics of shipped PANC-1 cells. (A) % RSD of the internal standards eluted alongside single cells in three batches with and without shipment (batch 1, *n* = 9; batch 2, *n* = 9; batch 3, *n* = 18, unshipped, *n* = 14). (B) mol % distribution of lipid classes of putatively identified lipids in 12 single PANC-1 cells. (C) Number of lipids detected vs their detection consistency across 12 PANC-1 cells. (D) Distribution of unique lipid identifications made in positive/negative ionization modes in 12 PANC-1 cells.

Figure 2A shows the relative standard deviation of the internal standard added to the lysis buffer and eluted from the capillary alongside the cell. The average RSD of all lipid classes was 13% for the samples that were not shipped, rising to 24% for the shipped samples. The increased variability is likely introduced through the drying and reconstitution procedure. Phosphatidylserine (PS) was the only lipid class that had considerable variability across all three shipped batches and was therefore excluded from the data set.

Figure 2B shows the average composition by lipid class of 12 internationally shipped single cells in mol %. This is similar to previously reported bulk measurement of a PANC-1 cell line,^22^ demonstrating that even after freeze-drying and international shipping, single-cell lipidomics can provide lipid profiles representative of bulk measurement.

Figure 2C shows the consistency of lipid species detection between cells. Of the 211 lipids identified across the experiment, 173 were detected in every cell. This shows a relatively low number of missing values and therefore a rich data set for each single cell with greater statistical power to compare groups. Furthermore, the total number of MS/MS confirmed identifications observed per single cell is consistent with both bulk and single-cell lipidomics reported previously for PANC-1 cells.^22–25^

Although the majority of lipid identifications were made in positive mode (Figure 2D), a sizable contribution (24%) was made from negative mode ionization. This demonstrates the utility of polarity switching to increase lipid coverage in single-cell samples. Furthermore, positive mode species can also be described with greater confidence as they have MS/MS spectra in both modes, providing information on the chain length and double bond position. An example spectrum of PC-O(34:2) obtained using polarity switching can be found in Supplementary Figure S3. The weighting toward positive mode identifications is likely due to the ammonium formate and formic acid buffer system, which favors positive ionization.^26^ In single-cell analysis, there is typically only enough material for a single injection; therefore, one buffer system must be used. Exploration of alternative buffer systems that have a better balance between positive and negative mode ionization could represent an opportunity to increase the coverage of single cell polarity switching experiments in future work.

### Discovery Group: Single-Cell Lipidomics Elucidates the Biological Impact of Radiation

Time course imaging was used to compare the formation of lipid droplets in irradiated and control (unirradiated) populations of cells. The greatest deviation between control and irradiated cells occurred 48 h after irradiation, and therefore cells were sampled for lipidomics analysis 48 h post-irradiation (Supplementary Figure S4). Figure 3 summarizes the single-cell lipidomics data for the discovery group (shipped cells). The global lipid profile between the control and radiation group can be separated by PLS-DA (Figure 3A), indicating broad differences in the lipid profile of the control and directly irradiated cells. Validation as determined by leave-one-out cross validation returned *R* ^2^ and *Q* ^2^ values of 0.95 and 0.71, respectively, for three components (see Supplementary Table S4), indicating good predictive accuracy and low number of outliers.^27^ Similarly, the control and bystander group can be separated by PLS-DA (Figure 3B) with *R* ^2^ and *Q* ^2^ values of 0.93 and 0.71,r espectively, for three components, again indicating significant differences in the lipid profile of the two groups. However, the PLS-DA for the bystander and radiation group (Figure 3C), although showing a tendency of separation, results in no significant separation of the two groups, with *R* ^2^ and *Q* ^2^ values of 0.88 and −0.10, respectively, for the three components. Others showed similar results when analyzing single cells infected with a parasite.^28^

**Figure 3:**
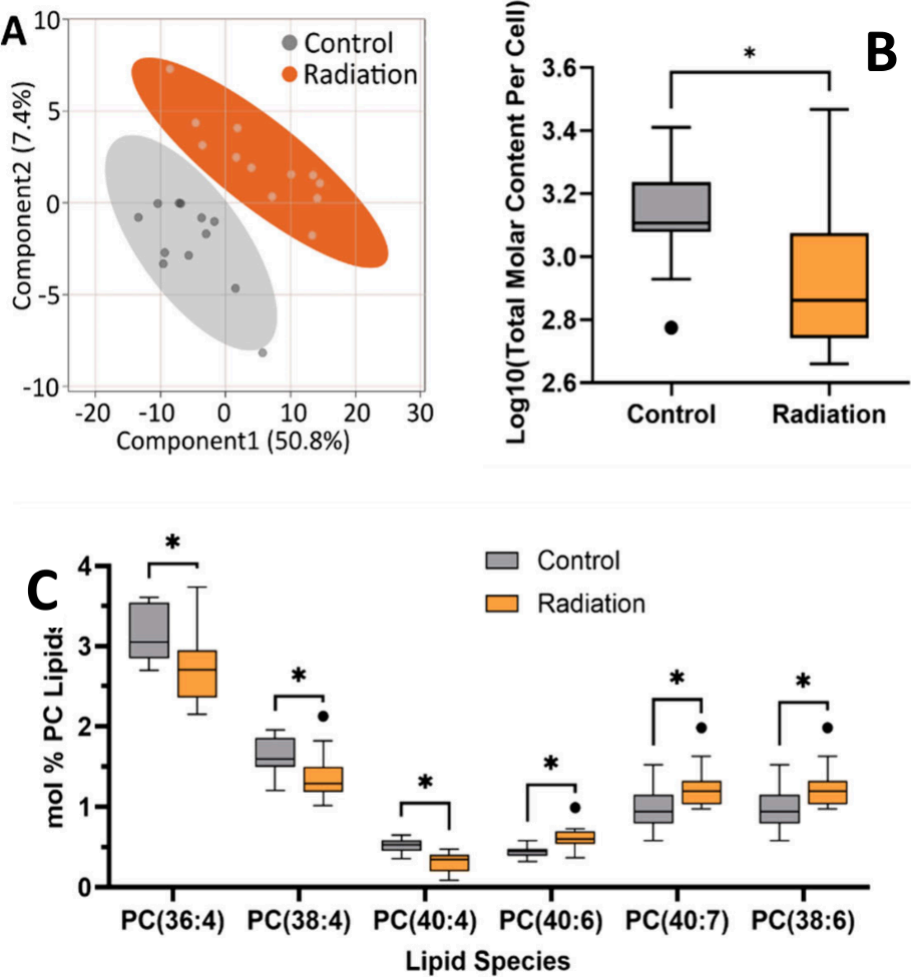
Lipid profile of shipped single PANC-1 cells. (A) PLS-DA plots of lipidomics output from control (*n* = 12) and directly irradiated (*n* = 12) cells. (B)­Total molar lipid content of control (*n* = 12) and directly irradiated (*n* = 12) cells. (C.) PC mol % of selected species containing arachidonic acid (20:4, ω-6) and docosahexaenoic acid (22:6, ω-3) in unirradiated controls (*n* = 12) and directly irradiated (*n* = 12) cells. *p < 0.05 as determined by two-tailed Mann–Whitney U test, **p < 0.01.

The total signal from all detected lipids (Figure 3B) reveals a distinctly lower abundance of lipids in cells which were directly irradiated (Mann–Whitney U, *n*_control_ = 12, *n*_radiation_ = 12, *U* = 28, *p* = 0.01). Similar trends in lipid abundance have been observed in bulk for other cell culture models of stress (including radiation) and are attributed to a decrease in *de novo* lipid synthesis.^29,30^ Other explanations for decreased lipid content include lipid catabolism to mitigate the impact of excess lipid peroxidation.^31^

It is noteworthy that many of the lipids, which were significantly lower in abundance in irradiated cells, contain polyunsaturated fatty acids. In particular, many species containing the fatty acid 20:4 (arachidonic acid) were impacted across multiple classes and with different neighboring chains (see Supplementary Table S5). The acquisition of negative mode phospholipid fragmentation allowed for the confirmation of the arachidonic acid moiety, further demonstrating the utility of polarity switching in single-injection samples. To understand changes in chain length and unsaturation in more detail, we compared the mol % within the PC lipid class (Table S6). A proportion of the PCs significantly impacted (Mann–Whitney U tests, *p* < 0.05) by direct irradiation were polyunsaturated. Of the 20 PC and O-PC species with a lower relative abundance in irradiated cells, 8 were lipids containing arachidonic acid. In contrast, 2 (out of 10) PC lipids increased in concentration and contained the ω-3 fatty acid docosahexaenoic acid (22:6, DHA), as shown in Figure 3C.

Polyunsaturated-containing phospholipids (PUFA-PLs) are crucial to radiation sensitivity through the process of ferroptosis, a nonapoptotic form of cell death mediated by iron metabolism and uncontrolled PUFA-PL peroxidation.^20^ Cells with lower concentrations of PUFA-PLs are more likely to survive oxidative stress following irradiation, as fewer PUFA chains undergo peroxidation and cause further cell damage.

The fluorescence data from the sampled cells are presented in Figures S6 and S7. Lipid droplets in directly irradiated cells were, on average, significantly larger in number and size than the control cells at both the population level and of the cells selected for single cell analysis. It is known that lipid membrane remodelling is required for the formation of lipid droplets.^32^ Enzymes which are involved in this remodelling to form lipid droplets are known to preferentially hydrolyze PUFA-PLs, therefore the decrease in PUFA lipids observed in the mass spectrometry data can be linked to the formation of lipid droplets observed in the microscopy. The decrease in abundance across many PUFA chains observed here from mass spectrometry data, combined with the formation of lipid droplets observed in the microscopy, indicates broad modulation of lipid metabolism to compensate for cellular stress of direct degradation following exposure to irradiation and subsequent generation of reactive oxygen species.

Cytosolic phospholipase A_2_α is an example of these enzymes and has selectivity for arachidonic acid-containing phospholipids.^33,34^ Free arachidonic acid, resulting from lipolysis, feeds directly into eicosanoid synthesis, with its downstream metabolites being typically pro-inflammatory. Anti-inflammatory eicosanoids are typically derived from free ω-3 fatty acids such as DHA.^35^ In the balance of pro- and anti-inflammatory eicosanoids, the trends observed in Figure 3C suggest catabolism of arachidonic acid-containing phospholipids and preservation of DHA-containing phospholipids across multiple phospholipid species, therefore likely presenting a pro-inflammatory state after radiation injury. Although the models are very different, parallel trends have been observed in mouse plasma 24 h after 8 Gy full body gamma irradiation.^17^

### Validation Group: Similarities in Lipid Dysregulation Following Irradiation Observed between Shipped (Discovery) And Unshipped (Validation) Cells

Figure 4 compares the data from the discovery group, where cells were freeze-dried and shipped internationally, to the validation group, where cells were simply stored at −80 °C before analysis. Of the 211 lipids detected in the discovery group, 103 were also detected in the validation group. This is due to the lower sensitivity of the validation method (which used analytical flow LC-MS) relative to the discovery method (which used nanoflow). Despite the increase in variability in internal standard response observed as a result of shipping (Figure 2A), Figure 4A shows that both the validation and discovery groups detect a similar relationship in total molar content of detected lipids between directly irradiated cells and controls.

**Figure 4:**
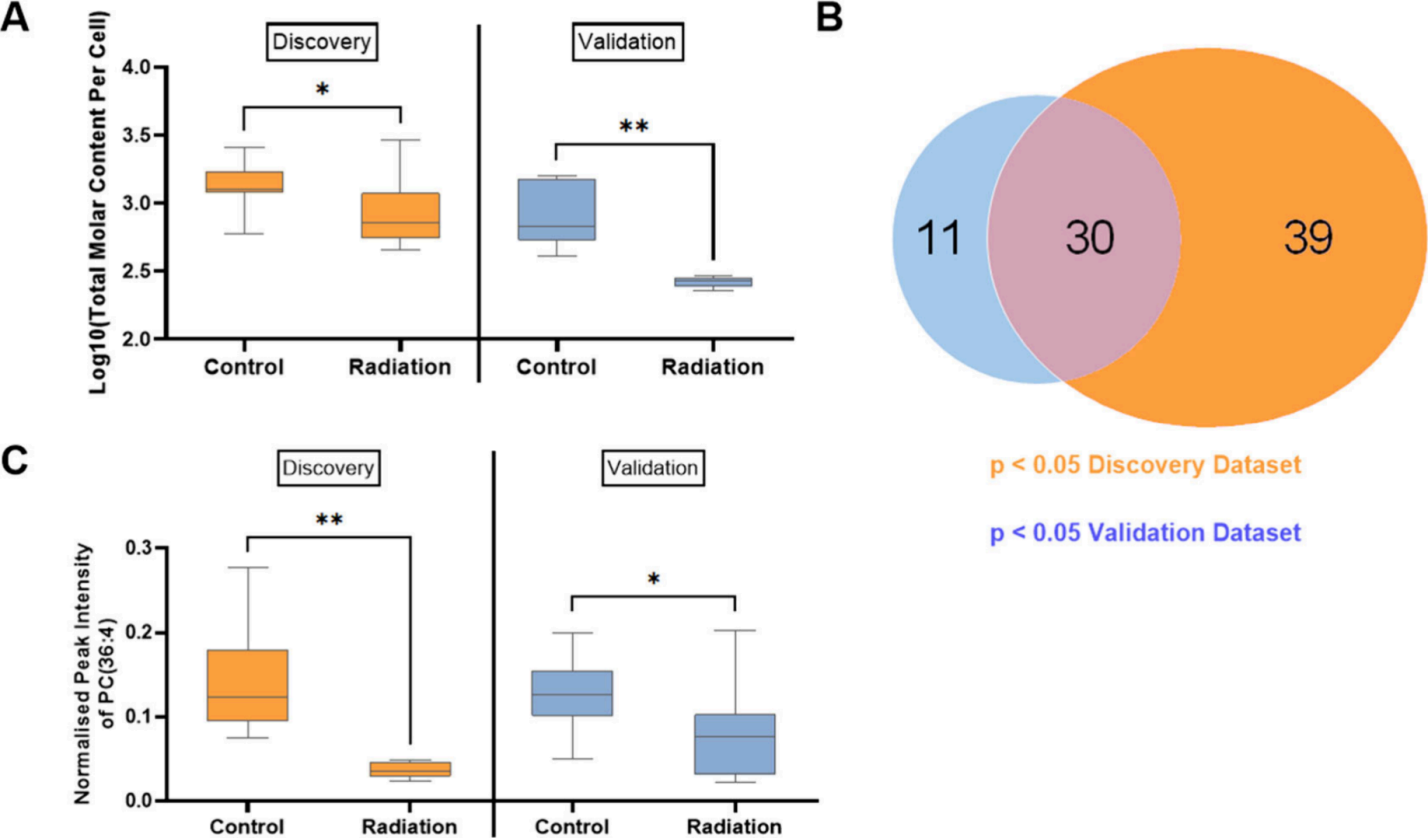
Lipid content of unshipped (validation) and shipped (discovery) single PANC-1 cells. (A) Total molar content of unshipped validation (control, *n* = 6; direct irradiation, *n* = 5) and shipped discovery cells (control, *n* = 12; direct irradiation, *n* = 12). (B) Overlap of significantly altered lipids between discovery and validation data sets. (C) Trends in PC(36:4) between unshipped validation (control, *n* = 6; direct irradiation, *n* = 5) and shipped discovery cell control (*n* = 12; direct irradiation, *n* = 12). *p < 0.05 as determined by two-tailed Mann–Whitney U test, **p < 0.01.

Figure 4B presents the lipids which differed in abundance (Mann–Whitney U, *p* < 0.05) between the control and directly irradiated cells, highlighting the similarities and differences between the discovery and validation data sets. 39 lipids were found to vary in the discovery data set only, and these were not systematically detected in the validation method, presumably due to a lack of sensitivity. Some lipids were found to vary only in the validation data set, and this can be explained by differences in measurement variability (reported in Figure 2A). What is striking is that although the (analytical flow) validation method detects fewer lipids, there is a 73% overlap with those significantly altered in the discovery data set.

Figure 4C demonstrates the similarity between the validation and the discovery group for PC(36:4), with similar findings across the other arachidonic acid-containing lipids (Supplementary Table S7). This demonstrates for the first time that biological effects on the lipidome of single cells can be detected by LC-MS, even when cells are sampled in one laboratory and shipped to another for analysis. The DHA-containing lipids highlighted in Figure 3C were also detected and tentatively identified in the validation cells analyzed by analytical flow LC-MS. However, in the analytical method, PC(38:6) showed no significant difference in terms of PC mol %, and PC(40:6) was only detected in a small number of cells. These lipids are naturally low in abundance, further highlighting the need for highly sensitive LC-MS/MS methodologies such as the nanoflow method used to measure the discovery group.

The single cells were shipped at room temperature in individual closed-capped vials under nitrogen atmosphere after freeze-drying to minimize oxidative and degradation effects on the lipid profile. It should be noted that previous work has shown changes to the metabolite profile of single cells after being freeze-dried and stored at −80 °C.^21^ Therefore, the absolute profile of metabolites in these samples might be expected to change as a consequence of freeze-drying and shipping. While the use of different platforms for the validation and discovery groups makes it difficult to decouple shipping effects from differences in instrument performance, the similarity in results observed between the two groups shows that any lipid changes do not preclude observation of a biological response after shipping. **The lipidomes of bystander and directly irradiated cells are indistinguishable yet distinctly different from those of unirradiated controls.**

“Bystander cells” were picked from areas shielded by a lead cover during irradiation (verified using Gafchromic film, Supplementary Figures S8 and S9). The positions of the bystander cells relative to the directly irradiated cells and irradiation zone are shown in Supplementary Figure S10. Figure 5 shows the comparison of bystanders with control and directly irradiated cells in terms of single-cell lipidomics output and live-cell imaging.

**Figure 5:**
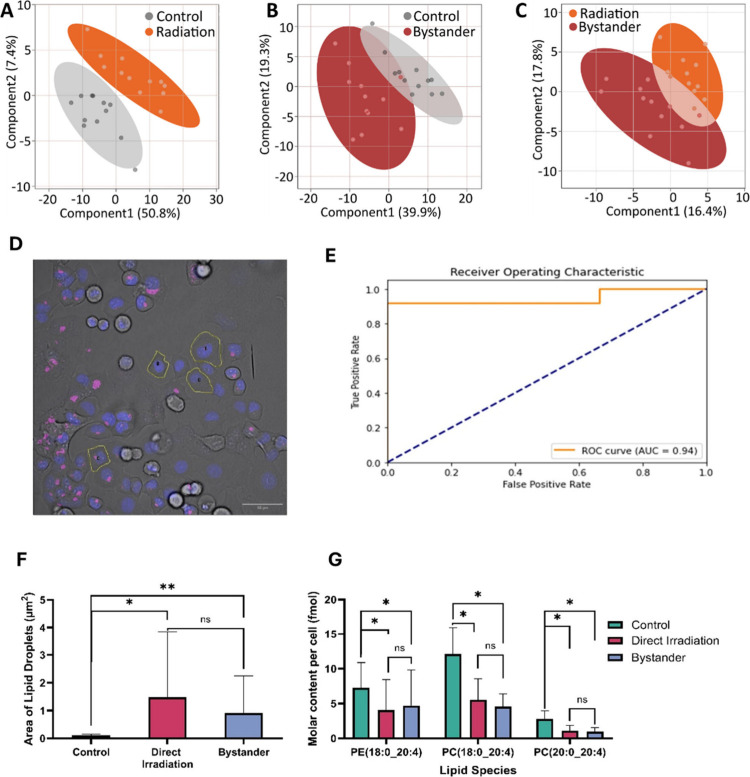
Lipid droplet profile of unshipped single PANC-1 cells. (A–C.) PLS-DA plots of lipidomics output from control (*n* = 12) directly irradiated (*n* = 12) and bystander (*n* = 12) cells. (A) Combined fluorescence (blue = Hoechst, pink = BODIPY 493/503) and brightfield channel image with overlay of cells isolated for lipidomics analysis (yellow). Image taken from population of bystander cells. Scale bar: 50 μm. (B) Receiver operating characteristic of the machine learning model used to characterize cells by treatment conditions (AUC = 0.94). (C) Lipid droplet area per cell between control (*n* = 12), directly irradiated (*n* = 12), and bystander cells (*n* = 12) directly sampled for lipidomics. (D) Trends in arachidonic acid-containing PC lipids between control (*n* = 12), directly irradiated (*n* = 12), and bystander cells (*n* = 12). *p < 0.05 as determined by two-tailed Mann–Whitney U test, **p < 0.01.

Figure 5A shows how the control and irradiated cells are separated using a PLS-DA model.

Data obtained from live-cell imaging (Figure 5F, exemplified in D) show that while the lipid droplet area of bystanders and directly irradiated cells is greater than the controls, there is no significant difference between bystanders and directly irradiated cells. This trend is repeated in images from the total population, both in lipid droplet size and count (Supplementary Figures S6 and S7). This suggests that signals have passed from directly irradiated cells via gap-junctions or through soluble factors in the media to bystander cells, altering the lipidome to resemble that of directly irradiated cells.^36–38^

Next, we applied a support vector machine (SVM) model to the mass spectrometry lipidomics data to distinguish between the control and irradiated cells. This gave 96% accuracy when tested by the leave one out cross-validation. The area under curve of 0.94 (Figure 5E) in the receiver operating characteristic curve indicates the excellent performance of the model in classifying cells into classes. When the model was used to test which class of features bystander cells were closest to, 11 out of 12 bystander cells were classed as irradiated. The lipids identified in bystander cells clearly differentiate from controls for most examples but fail to differentiate from the directly irradiated cells. Figure 5F illustrates this for three arachidonic acid containing phospholipids.

These results effectively demonstrate that the bystander PANC-1 cells, which were in close proximity to irradiated cells for 48 h, could not be distinguished from the directly irradiated cells which had survived treatment. To the best of our knowledge, this is the first work to show how changes to lipids manifest spatially after irradiation. The lipid profiles of both directly irradiated cells and their bystanders were significantly altered compared to those of the control cells, which received no dose. Recent publications suggest that the lipid content of cancer cells directly impacts their resistance to treatment with either chemotherapy or radiotherapy.^31,39^ The data integrated from both live-cell imaging and single-cell lipidomics in this work suggest that alterations are also made to in polyunsaturated lipid metabolism of bystanders, which are not directly hit by radiation. It is understood that cells which survive irradiation become subsequently more resistant to treatment, but the exact mechanism of radioresistance is not yet known.^40^ The lipidomic alterations of bystander cells and similarity to the directly irradiated cells could suggest an increase in resistance, specifically through the downregulation of peroxidation-susceptible PUFA-phospholipids. These findings therefore highlight the important potential for using single-cell lipidomics to optimize targeting and dose delivery of radiation to cancerous tissue, mitigating the impact of surviving cells and their local influence on cancer cells missed by the dose.

While this work yields novel insight into the single-cell lipidome of pancreatic cancer postirradiation, it also highlights future opportunities for the development of single cell multiomics. It is well established that the overexpression of COX-2, which is downstream of PUFA-phospholipid catabolism, is implicated in cancer treatment resistance through overproduction of arachidonic acid-derived eicosanoids.^41,42^ The observation of decreased PUFA-lipids are consistent with this understanding of eicosanoid metabolism through the lens of phospholipid catabolism, especially as COX-2 has been implicated in the radiation induced bystander effect in transgenic mice models.^43^ Future work should aim to incorporate proteins and transcripts from the same cells, potentially yielding a comprehensive mechanistic understanding of the radiation model described and providing greater insights into potential therapeutic targets. Additionally, future work should explore how lipid variations manifest at different distances between irradiated cells and their bystanders.

## Supplementary Material





## Data Availability

The raw data can be accessed at Zenodo Repository: 0.5281/zenodo.15673516.

## Notes

Lipidomics data from LC-MS/MS analysis (XLSX)
Comparison of dyed vs control PANC-1 cells; example mass spectrum for polarity switching; 48 h lipid droplet analysis; Venn diagram of control, radiation, and bystander cells; X-ray irradiation setup; LC-MS/MS parameters; and PLS-DA cross-validation (PDF)
