# Supplementary material for: Single-Cell Lipidomics by LC-MS Interlaboratory Study Reveals the Impact of X‑ray Irradiation on a Pancreatic Cancer Cell Line and Its Bystanders
Source: Anal Chem. 2025 Jun 18;97(25):13532–41. doi: 10.1021/acs.analchem.5c02010 (PMC12224164; doi:10.1021/acs.analchem.5c02010)
Supplement: Supplementary file 2 [file ac5c02010_si_002.pdf]

Supporting Information:

Single-cell lipidomics by LC-MS inter-laboratory study reveals the impact of X-ray irradiation on a pancreatic cancer cell line and its bystanders

Kyle D. G. Saunders[1], Johanna von Gerichten[1], Rahul Deshpande[2], Matt Spick[3], Susan Bird[2], Giuseppe Schettino[4], Hannah Bolland[5], Anthony Whetton,[5] Eirini Velliou[6], Melanie Bailey[7,1]*

[1] School of Chemistry and Chemical Engineering, Faculty of Engineering and Physical Sciences, University of Surrey, Guildford, GU2 7XH, UK

[2] Thermo Fisher Scientific, 355 River Oaks Parkway, San Jose, CA 95134, United States.

[3] School of Health Sciences, Faculty of Health and Medical Sciences, University of Surrey, Guildford, GU2 7XH, UK

[4] National Physical Laboratory, Hampton Rd, Teddington, TW11 0LW, U.K.

[5] School of Veterinary Medicine, Faculty of Health and Medical Sciences, University of Surrey, Guildford, GU2 7XH, UK

[6] University College London, Division of Surgery and Interventional Science, London W1W 7TY, UK

[7] Department of Infectious Diseases, Guy's Hospital, King's College London, SE1 9RT, London, UK

***Corresponding author email**: melanie.j.bailey@kcl.ac.uk

Table of Contents:

Cover page and table of contents 1-2
Figure S1 PCA dyed vs control single PANC-1 cells 3
Figure S2 Mann-Whitney U test dyed vs control PANC-1 cells..7
Figure S3 Mass spectrum of PC-O(34:2) in positive and negative mode 4
Figure S4 48 hours lipid droplet analysis via fluorescence imaging....................................4
Figure S5 Venn diagram for control, radiation and bystander group...................................4
Figure S6 Average lipid droplet size in cells..5
Figure S7 Number of lipid droplets per cell 5
Figure S8 Cell radiation with lead shielding 6
Figure S9 Gafchromic film for radiation area..6
Figure S10 Single cell selection of radiation and bystander group 6
Table S1 LC-MS/MS parameter 7
Table S2 Acquisition method parameters used for the nanoflow method 8
Table S3 Acquisition method parameters used for the analyical flow method 8
Table S4 Leave one out cross validation results for PLS-DA..8

Cell dye impact assessment

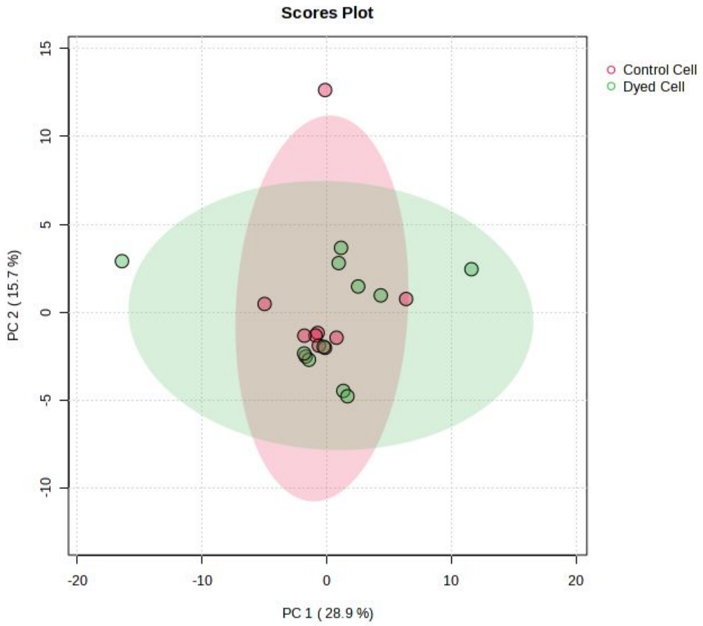

Supplementary Figure S1 *Principal components analysis (PCA) of lipid profiles from dyed single cells (green, n = 11) vs control cells (red, n = 10).*

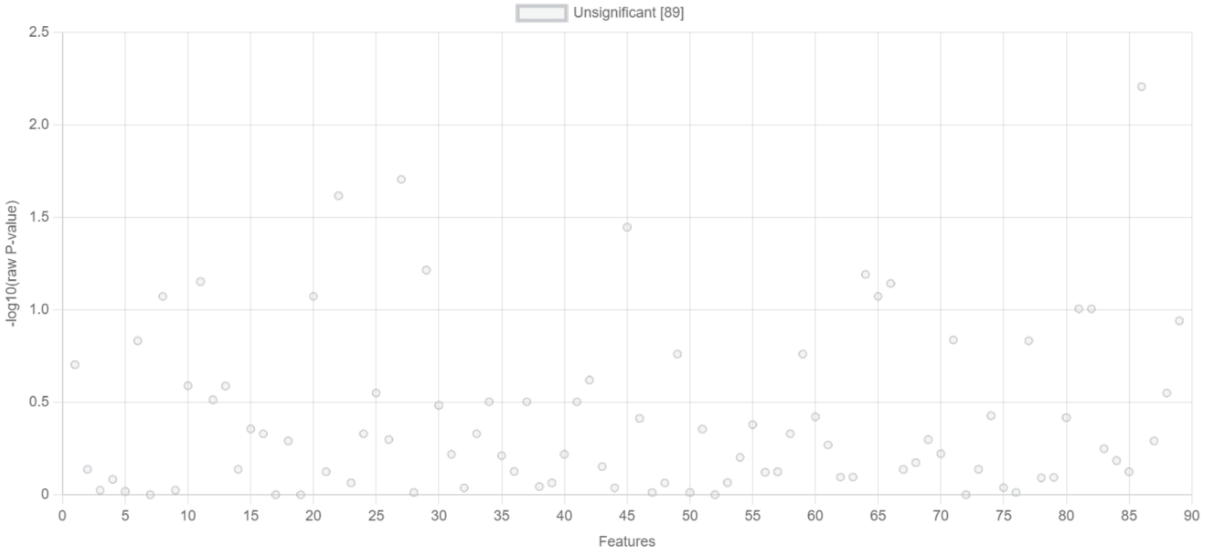

Supplementary Figure S2 *Mann-Whitney U significance test results for each lipid identified between dyed cells (n = 11) and undyed control cells (n = 10). Significance limit set to p = 0.05.*

Polarity switching example spectra

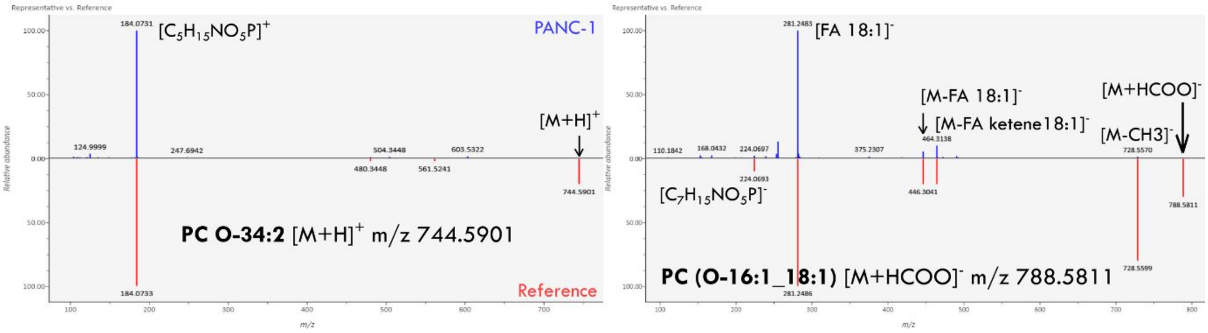

Supplementary Figure S3 *Example spectra of PC-O(34:2) measured from a single cell using polarity switching on the Exploris 240. PC-O(36:2) is determined by PC headgroup and detection of M+H ion in positive mode. At the same retention time, negative mode describes the fatty acid composition of 18:1, allowing for deduction of 16:1.*

X-Ray timelapse imaging

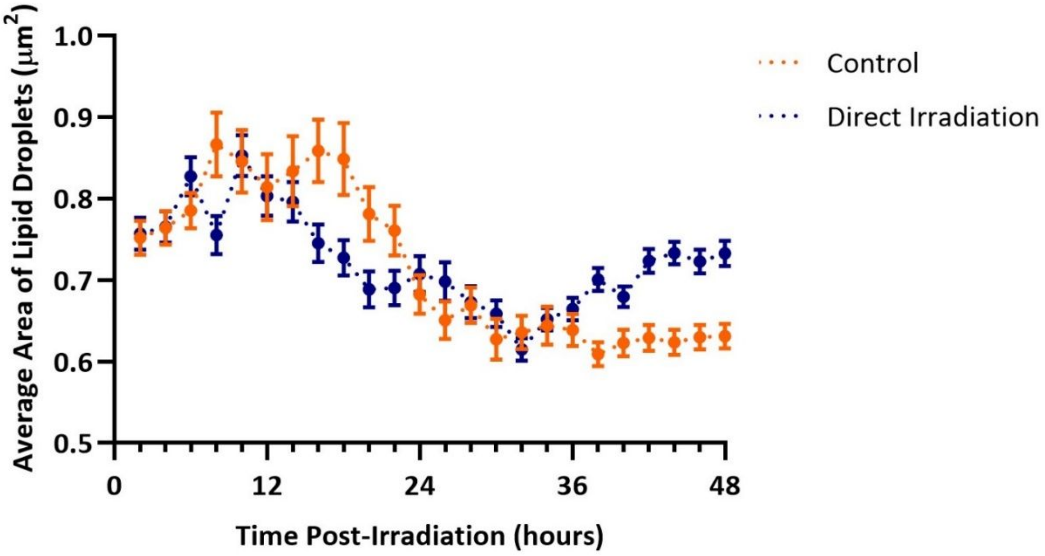

Supplementary Figure S4 *Timelapse of lipid droplet area (μm^2) over 48 hours post 6 Gy X-ray irradiation vs control with fluorescence imaging of cells dyed with Hoechst and BODIPY. Error bars = ± 1 sd.*

Fluorescence imaging of lipid droplets

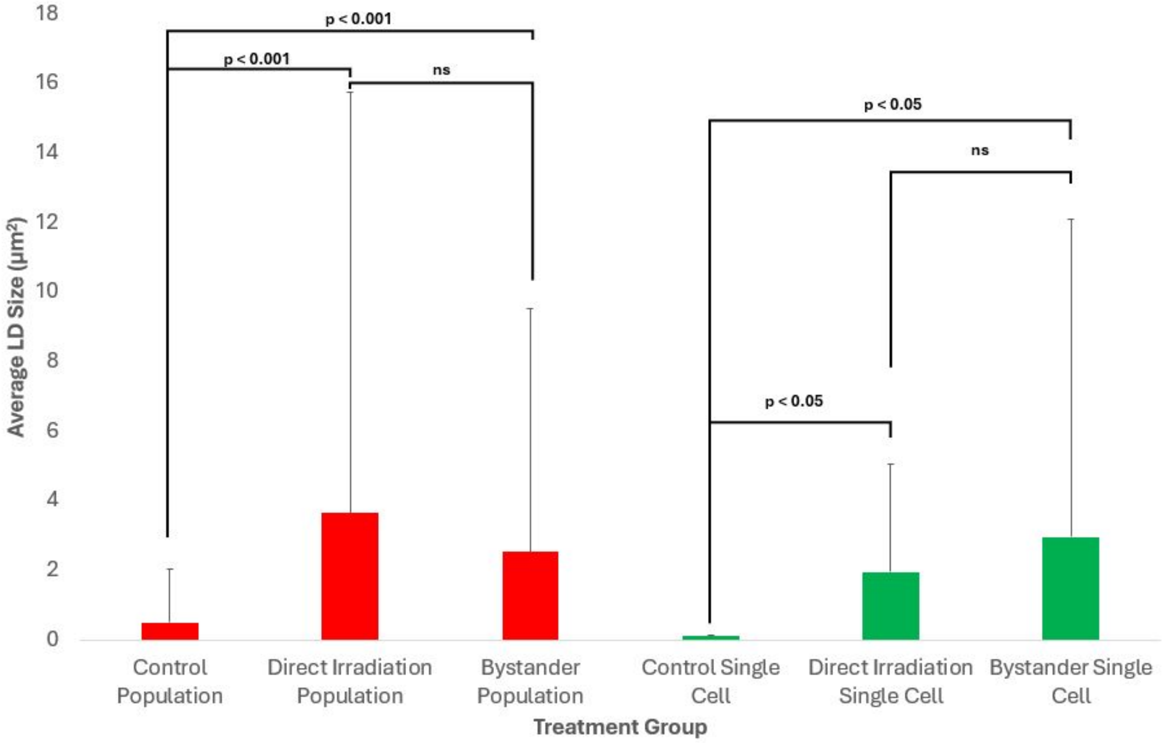

Supplementary Figure S6 *Lipid droplet size for each condition at the population level vs sampled single cells. Population Control n = 169, Population Direct Irradiation n = 279, Population Bystander n = 241, Single Cell Control n = 5, Single Cell Direct Irradiation n = 19, Single Cell Bystander n = 59. Error bars = 1 sd. Mann-Whitney U test used for significance testing.*

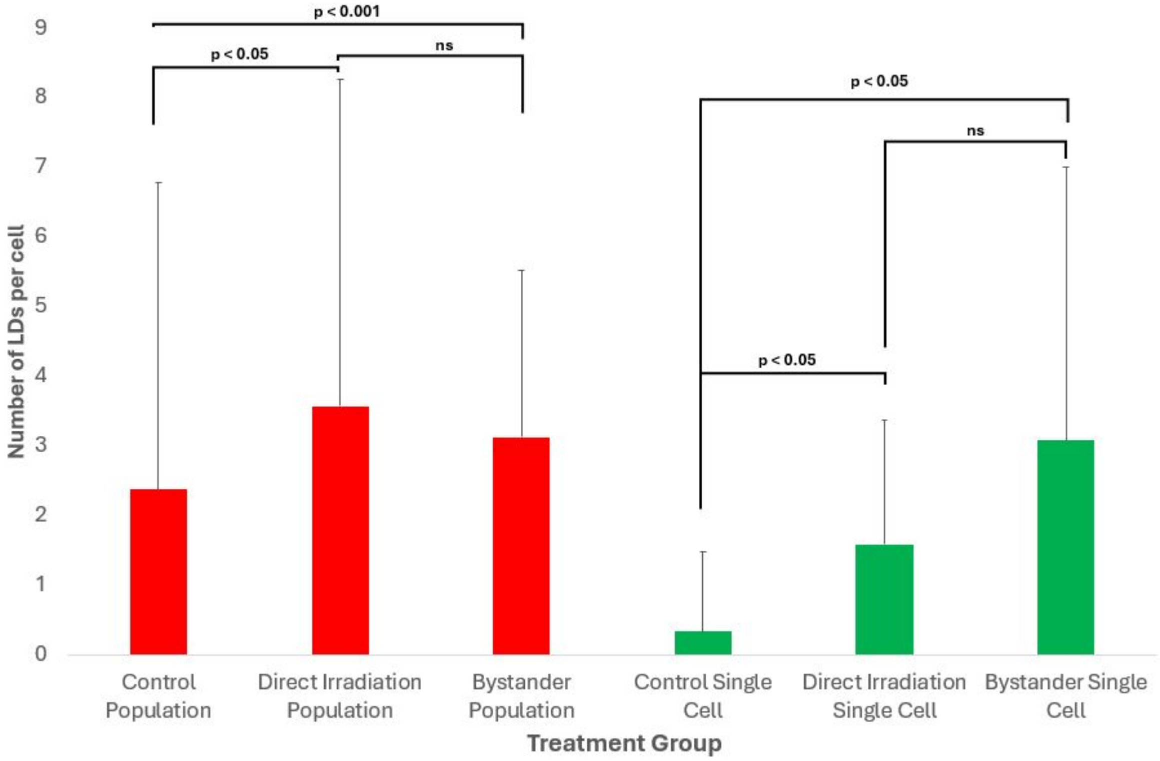

Supplementary Figure S7 *Number of lipid droplets per cell for each condition at the population level vs sampled single cells. Population Control n = 71, Population Direct Irradiation n = 78, Population Bystander n = 77, Single Cell Control n = 12, Single Cell Direct Irradiation n = 12, Single Cell Bystander n = 12. Error bars = 1 sd. Mann-Whitney U test used for significance testing.*

Cell irradiation apparatus and sampling coordinates

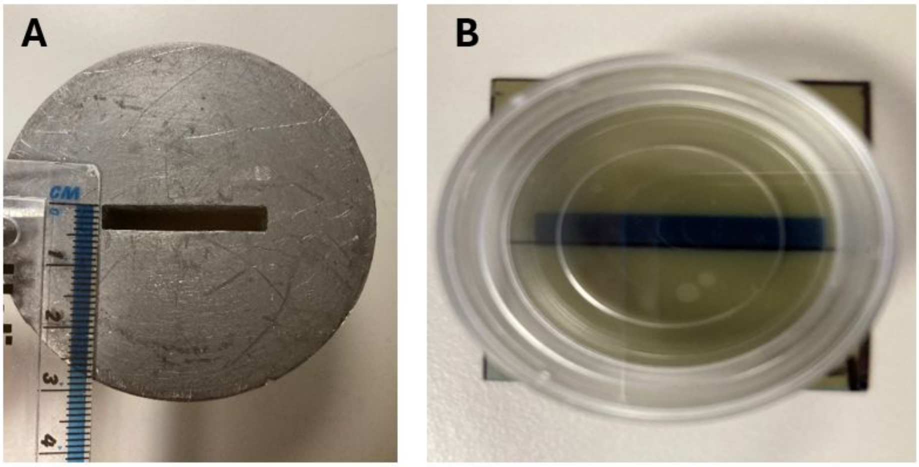

Supplementary Figure S8 *- A.) Lead shielding used to protect cell culture with defined 4 x 21 mm slit with 21 mm thickness. B.) Petri dish used for cell culture and irradiation imposed on top of EBT3 Gafchromic film following 6 Gy irradiation, indicating area of irradiation. Sampling was only possible in central circle.*

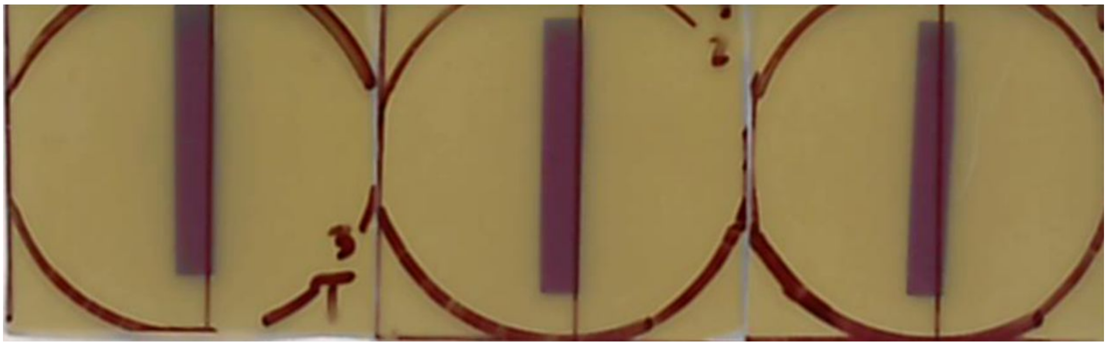

Supplementary Figure S9 *Three replicate EBT3 Gafchromic film samples placed underneath media-containing (2 mL) petri dish and lead shielding. The projected outer circumference of the petri dishes are marked in pen.*

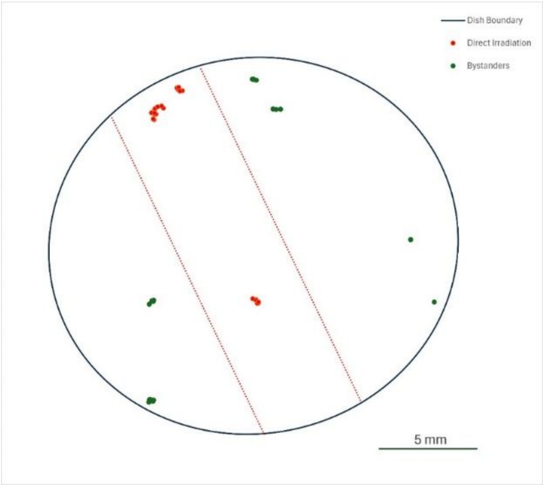

Supplementary Figure S10 *- Projected coordinates of isolated single cells relative to the irradiated zone. Scale bar = 5 mm.*

LC-MS/MS parameters

Supplementary Table S1 *Comparison of chromatographic parameters between analytical and nano flow methods used for single cell analysis.*

<table>
<tr><th>Parameter</th><th>Analytical Flow</th><th>Nanoflow</th></tr>
<tr><td>Instrument</td><td>Thermo Scientific™ UltiMate™ 3000 UHPLC
Thermo Scientific QExactive™ Plus</td><td>Thermo Scientific™ Vanquish™ Neo
Thermo Scientific Exploris™ 240</td></tr>
<tr><td>Column</td><td>Thermo Scientific Accucore C30
(2.6 µm, 2.1 x 150 mm)</td><td>Thermo EASY-Spray™ PepMap™
(75 µm x 15 cm)</td></tr>
<tr><td>Solvent A</td><td>60:40 (ACN/H_2O)
+ 0.1 % formic acid
+ 10 mM ammonium formate</td><td>60:40 (ACN/H_2O)
+ 0.1 % formic acid
+ 10 mM ammonium formate</td></tr>
<tr><td>Solvent B</td><td>85:10:5 (IPA / H_2O / ACN)
+ 0.1 % formic acid
+ 10 mM ammonium formate</td><td>88:10:2 (IPA / CAN / H_2O)
+ 0.1 % formic acid
+10 mM ammonium formate</td></tr>
<tr><td>Temperature</td><td>40 °C</td><td>45 °C</td></tr>
<tr><td>Injection Volume (µL)</td><td>15</td><td>12</td></tr>
<tr><td>Flow Rate</td><td>350 µL/min</td><td>750 nL/min</td></tr>
<tr><td>Gradient</td><td>
<table>
<tr><th>Time (mins)</th><th>% A</th><th>% B</th></tr>
<tr><td>0.0</td><td>70</td><td>30</td></tr>
<tr><td>5.0</td><td>70</td><td>30</td></tr>
<tr><td>5.1</td><td>57</td><td>43</td></tr>
<tr><td>14</td><td>30</td><td>70</td></tr>
<tr><td>14.1</td><td>30</td><td>70</td></tr>
<tr><td>21</td><td>1</td><td>99</td></tr>
<tr><td>24</td><td>1</td><td>99</td></tr>
<tr><td>24.1</td><td>70</td><td>30</td></tr>
<tr><td>28</td><td>70</td><td>30</td></tr>
</table>
</td><td>
<table>
<tr><th>Time (min)</th><th>% A</th><th>% B</th></tr>
<tr><td>0</td><td>70</td><td>30</td></tr>
<tr><td>1</td><td>70</td><td>30</td></tr>
<tr><td>3</td><td>45</td><td>55</td></tr>
<tr><td>5</td><td>25</td><td>75</td></tr>
<tr><td>9</td><td>5</td><td>95</td></tr>
<tr><td>12</td><td>0</td><td>100</td></tr>
<tr><td>16</td><td>0</td><td>100</td></tr>
<tr><td>16.2</td><td>70</td><td>30</td></tr>
<tr><td>20</td><td>70</td><td>30</td></tr>
</table>
</td></tr>
</table>

Supplementary Table S2 *Acquisition method parameters used for the nanoflow method (discovery dataset).*

Parameters	*Full MS*	*dd-MS²*
Instrument	Thermo Scientific Exploris™ 240	
Polarity	Positive and Negative (Polarity Switching)	
Spray Voltage (V)	Positive = 2500, Negative = 2000	
Resolution	60,000	15,000
AGC Target (normalised, %)	100%	Default
Maximum Injection Time (ms)	100	100
Scan Range (m/z)	250 - 1250	
Isolation Window (m/z)		1.4
Collision Energy (normalised to m/z 500, z =1)		35
Minimum AGC Target (for MS/MS triggering)		Default
Dynamic Exclusion Window (s)		3

Supplementary Table S3 *Acquisition method parameters used for the analytical flow method (validation dataset).*

Parameters	*Full MS*	*dd-MS²*
Instrument	Thermo Scientific QExactive™ Plus	
Polarity	Positive	
Resolution	70,000	17,500
AGC Target	$1e^6$	$5e^5$
Maximum Injection Time (ms)	400	50
Scan Range (m/z)	200 – 1200	200-1200
Isolation Window (m/z)		4.0
Collision Energy (eV)		20
Minimum AGC Target (for MS/MS triggering)		$2.00\ e^3$
Dynamic Exclusion Window (s)		6

Control vs direct irradiation: Partial least squares discriminant analysis validation

Supplementary Table S4 *Leave one out cross validation results for PLS-DA model generated for discovery single cell dataset, direct irradiation vs control.*

Measure	1 comps	2 comps	3 comps	4 comps	5 comps
Accuracy	0.75	0.91667	0.91667	0.91667	0.91667
R2	0.41306	0.90116	0.94647	0.96981	0.98869

Q2	0.25873	0.57712	0.71086	0.68551	0.67925
